# Magnetic resonance findings of Stewart–Treves Syndrome in primary limb lymphedema compared with pathology: A retrospective single-center study

**DOI:** 10.3389/fonc.2023.953524

**Published:** 2023-02-09

**Authors:** Bin Li, Jiyuan Li, Kun Hao, Yanfang Jin, Jun Ma, Xuemei Du

**Affiliations:** ^1^ Department of MRI, Beijing Shijitan Hospital, Capital Medical University, Beijing, China; ^2^ Department of Lymphatic Surgery, Beijing Shijitan Hospital, Capital Medical University, Beijing, China; ^3^ Department of Radiology, Chuiyangliu Hospital Affiliated to Tsinghua University, Beijing, China; ^4^ Department of Pathology, Beijing Shijitan Hospital, Capital Medical University, Beijing, China

**Keywords:** Stewart-Treves Syndrome, MRI, primary, lymphedema, low limb

## Abstract

**Background:**

Stewart–Treves Syndrome in Primary Limb Lymphedema (STS-PLE) is an extremely rare malignant tumor. A retrospective analysis was conducted to elucidate the relationship between magnetic resonance imaging (MRI) findings and signs compared to pathology.

**Methods:**

Seven patients with STS-PLE were enrolled at Beijing Shijitan Hospital, Capital Medical University, from June 2008 to March 2022. All cases were examined by MRI. The surgical specimens were subjected to histopathological and immunohistochemical staining for CD31, CD34, D2-40, and Ki-67.

**Results:**

There were two different types of MRI findings. One was mass shape (STS-PLE I type) in three male patients, and the other was the “trash ice” d sign (STS-PLE II type) observed in four female patients. The average duration of lymphedema (DL) of STS-PLE I type (18 months) was shorter than that of STS-PLE II type (31 months). The prognosis for the STS-PLE I type was worse than that for the STS-PLE II type. Regarding overall survival (OS), the STS-PLE I type (17.3 months) was three times shorter than that of the STS-PLE II type (54.5 months). For STS-PLE I type, the older the STS-PLE onset, the shorter the OS. However, there was no significant correlation in STS-PLE II type. MRI was compared to histological results to provide an explanation for the differences in MR signal changes, especially on T2WI. Against a background of dense tumor cells, the richer the lumen of immature vessels and clefts, the higher the T2WI MRI signal (taking muscle signal as the internal reference standard) and the worse the prognosis, and vice versa. We also found that younger patients with a lower Ki-67 index (<16%) had better OS, especially for the STS-PLE I type. Those with stronger positive expression of CD31 or CD34 had shorter OS. However, the expression of D2-40 was positive in nearly all cases, and seemed not to be associated with prognosis.

**Conclusions:**

In lymphedema, the richer the lumen of immature vessels and clefts based on dense tumor cells, the higher the T2WI signal on the MRI. In adolescent patients, the tumor often showed a “trash ice” sign (STS-PLE II-type) and prognosis was better than for the STS-PLE I type. While in middle-aged and older patients, tumors showed a mass shape (STS-PLE I type). The expression of immunohistochemical indicators (CD31, CD34, and KI-67) correlated with clinical prognosis, especially decreased Ki-67 expression. In this study, we determined it was possible to predict prognosis comparing MRI findings with pathological results.

## 1 Introduction

Stewart–Treves Syndrome (STS) is as an aggressive malignant tumor with a poor prognosis (1). To date, only a few cases have been reported (2). The majority of reported cases were the result of postoperative lymphedema, mainly (90%) after mastectomy (3–5). In clinical practice, STS has often been misdiagnosed and underestimated due to the following factors (6–8): 1) abnormal skin thickening caused by fibrous hyperplasia and hyperkeratosis related to chronic lymphedema, 2) STS lesions mimicking benign tumors with a clear outline, and 3) difficulty distinguishing STS lesions latently distributed in the subcutaneous tissue where lymphedema occurs.

To our knowledge, magnetic resonance imaging (MRI) findings of STS-Primary Limb Lymphedema (STS-PLE) have not been reported, especially in cases involving the lower limb. Moreover, a systematic assessment of STS is lacking. In this retrospective study, a systematic analysis that included clinical and pathological data was performed to investigate MRI findings in the diagnosis and evaluation of STS-PLE.

## 2 Methods

### 2.1 Ethical approval

The study was approved by the Human Research Ethics Committee of the Shijitan Hospital of Capital Medical University of Beijing. Written informed consent was obtained from the individuals(s) for the publication of any identifiable images or data included in the article. All procedures were conducted in accordance with the ethical standards of human experimentation (institutional or regional) and with the Helsinki Declaration of 1975, as revised in 2000. Because this was a retrospective study and the data analysis was performed anonymously, this study was exempted from ethical approval and informed consent from each patient.

### 2.2 Patient population

Between June 2008 and March 2022, a total of 9831 cases of limb lymphedema (age range, 1–78 years; mean ± standard deviation, 48.0 ± 31.7 years) were diagnosed at Beijing Shijitan Hospital. In total, seven STS-PLE patients (age range, 15–66 years; median age 39 years) were clinically and pathologically verified. The following clinical data were collected: duration of lymphedema (LD), specific skin findings (appearance, color, temperature, ulcer and hemorrhagic discharge, and surgical methods), and survival time.

### 2.3 Magnetic resonance imaging

MRI was performed with a 1.5T MRI unit (Ingenia, Philips Medical Systems, The Netherlands) using a 16-channel body coil. The checked limbs were placed in the correct anatomical location and as centered in the coil as possible for the patient. MRI was performed with the following sequences: STIR/axial and coronal planes (TR 5200 ms, TE 80 ms IT 160 ms, Slice 5 mm, GAP 0.5 mm, Voxel 1.5 mm^2^, FOV 220 mm×200 mm/320 mm×240mm, NEX=2) and mDixon (TR 5.5 ms, TE_1 =_ 1.8 ms, TE_2 =_ 4.0 ms, Slice 5 mm, GAP 0, Voxel 1.5 mm^2^, and FOV 220 mm×200 mm/320 mm×240 mm, NEX=2).

To determine infiltrative growth (6), a supplementary explanation of the “trash ice” sign was established, which refers to the appearance of scattered patches, with intermediately low and heterogeneous signals on T2WI, against the background of lymphedema without recognizable signs of it (6, 7). All MRI images were interpreted by two lymphedema radiologists who had 5 and 10 years of experience, respectively. The MRI findings including location (derma, subcutaneous tissue, and superficial fascia of muscle), shape, outline, and signal intensity of STS-PLE lesions were identified and analyzed, based on the consensus of two radiologists.

### 2.4 Histopathological analysis

Resected surgical specimens were fixed in 10% phosphate-buffered, neutral formaldehyde solution at room temperature for 24 h and dehydrated in an ascending series of ethanol. The samples were routinely embedded in paraffin, made transparent with xylene, rehydrated in a descending series of ethanol, washed with distilled water, and stained with hematoxylin and eosin (HE) for 30 min at room temperature (VENTANA HE600 System, Roche). Sections (4 µm thick) were observed under a light microscope (ZEISS Axio Scope.A1) with magnifications of ×40, ×100, ×200, and ×40.

### 2.5 Immunohistochemistry

Tissue sections (4 µm thick) were deparaffinized, rehydrated, and subjected to antigen retrieval with FLEX High Ph Target Retrieval solution High Ph (50×) according to the manufacturer’s protocol (EnVision FLEX+, Mouse, high Ph (Link) HRP; cat. no. K8002; Dako; Agilent Technologies, Inc., Santa Clara, CA, USA) in PT Link (cat. number PT100; Dako; Agilent Technologies, Inc.) at 95°C for 20 min and washed in distilled water (9). Endogenous peroxidase was blocked by DAKO Envision flex peroxidase blocking reagent for 10 min and washed again three times in PBS wash buffer (Origene Technologies, Inc., Wuxi, China). All slides were incubated for 20–30 min at room temperature in a humidity chamber with appropriate dilutions of primary antibodies (**Table 1**).

**Table 1:** Primary antibodies used for immunohistochemistry.

Target	Supplier	Catalog number	Dilution
CD31	Origene Technologies, Inc.	ZA-0568	Ready to use
CD34	Origene Technologies, Inc.	ZA-0550	Ready to use
D2-40	Gene Tech Co., Ltd.	GM361929	1:60
Ki67	Origene Technologies, Inc.	UM870033	1:100

The sections were then incubated with secondary antibody (MA-2000, Origene Technologies, Inc., Wuxi, China) for the coupling reaction for 20–30 min at room temperature. The substrate (EnVision FLEX DAB+ Chromogen) was used to produce a crisp brown color at the target antigen. Hematoxylin (1 or 2 dips) was used as a counter stain.

The sections were observed under a light microscope with magnifications of ×40, ×100, ×200, and ×400. Two senior pathologists independently scored immunohistochemical staining. The number of stained nuclei was expressed as a percentage (index) of positively immunoreactive cells. The proportion of Ki-67 positive cells was counted in at least 1000 tumor cells in the highest labeled area (defined as the hot spot) (10).

## 3 Results

### 3.1 Clinical data

STS-PLE was detected in 0.07% (7/9831) of the study population. The average LD, median survival time (MST), and 5-year survival rate were 25.4 years, 45 months, and 28.6% (2/7), respectively. Of the seven cases (**Table 2**), the primary locations of STS-PLE were the following: thigh in one case, hand in one case, ankle in two cases, and foot in three cases. The ratio of the left to the right limb was 4:3. Negative skin findings were observed in one young female patient, but positive in the other three female patients. Three male patients presented positive findings of all four items. The ratio of wide excision to amputation was 4:3, in which OS of the former (34.75 months) was nearly 9 months less than the latter (43.67 months).

**Table 2:** Clinical characteristics of the patients with STS-PLE.

Number	Sex	Age (years)	DL^*^ (years)	Side/Location	Skin findings^†^ Protuberance	color^‡^	temperature ulcer	HD	Surgical method^§^	Survival (months)
1	Male	63	6	Right/thigh	1	dark red	1	1	A	2
2	Male	39	25	Left/foot	1	dark red	1	1	A	5
3	Male	23	23	Left/hand	1	dark red	1	1	B	45
4	Female	37	37	Right/foot	1	dark red	1	0	B	26
5	Female	15	6	Left/ankle	1	dark red	1	0	A	60
6	Female	21	21	Left/ankle	0	0	0	0	A	72^||^
7	Female	66	60	Right/foot	1	purple	1	0	B	60 ¶

* Calculation method in duration of lymphedema: starting point is the year of diagnosis of limb lymphedema, end point is the year of diagnosis of STS, taking the whole year as the counting unit. †, 0, negative; 1, positive. ‡, the color of spot in the skin. §, A represents wide resection; B represents amputation. ||, alive with lung metastasis. ¶, alive with lung metastasis and bone metastasis. HD refers to hematological discharge.

The male to female ratio was 3:4. The average LD (18 months) of three male patients was shorter than that of four female patients (31 months). The average survival time in male patients (17.3 months) was three times shorter than in female patients (54.5 months). For male patients, the older the STS-PLE onset, the shorter the survival. However, there were no significant differences in female patients (**Table 3**).

**Table 3:** MR findings of the patients with STS-PLE.

Number	Shape	Outline	T1WI^**^	T2WI^††^	Scope of invasion
1	Mass	Unclear	Moderate	Heterogeneous	1.2
2	Mass	Unclear	Slight	Heterogeneous	1.2.3
3	Mass	Unclear	Slight	Heterogeneous	1.2.3
4	Trash Ice	Unrecognized	Moderate	Intermediate	1.2
5	Trash Ice	Unrecognized	Moderate	Intermediate	1.2
6	Trash Ice	Unrecognized	Moderate	Intermediate	1.2
7	Trash Ice	Unrecognized	Moderate	Intermediate	1.2

**The muscle signal in the same layer was taken as medium signal of the internal reference. †† 1 represents skin and dermis, 2 represents subcutaneous tissue, and 3 represents superficial fascia.

### 3.2 Magnetic resonance findings

**Figure 1:**
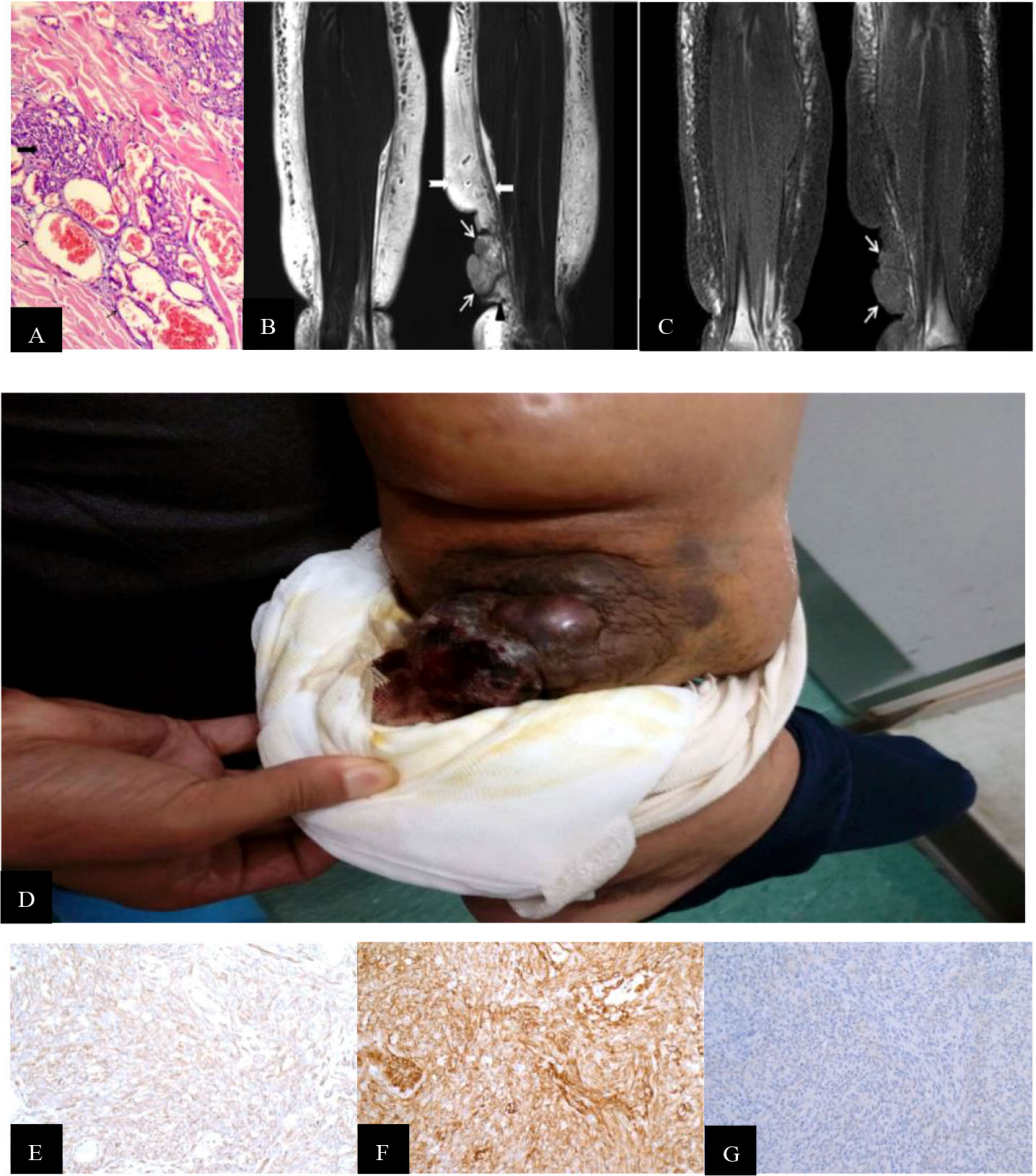
**(A)** Hematoxylin-and-eosin-stained histopathology of the M200 STS-PLE I type: the tumor nests were composed predominantly of densely atypical epithelioid cells displaying hyperchromatism (wide black arrowhead). The spindle endothelial cells lined immature vessels, with erythrocytes detected inside the vessels (fine black arrowhead) and distributed in collagen fiber stroma (+). **(B)** Coronal T2WI identified a mass-like shape (fine white arrowhead) located in the subcutaneous soft tissue of the left medial malleolus. Its signal was heterogeneously decreased against the background of lymphedema. If compared with normal muscle, the tumor signals are increased heterogeneously on the T2WI. The extensive lymphedema (swallow-tail white arrowhead) is indicated by an abnormally thickened superficial fascia (wide white arrowhead) and adjacent thickened skin. **(C)** Corresponding T1WI signal of STS was slightly decreased compared with the normal muscle (fine white arrowhead). **(D)** A large mass was located and protruded in the surface of the lower leg, and presented as a dark red superficial ulcer with hemorrhagic discharge, dark-red spots, and increased skin temperature. **(E, F)** CD31 and CD34 staining were positive (magnification, x200). **(G)** D2 40 staining was partially positive (magnification, x200).

**Figure 2:**
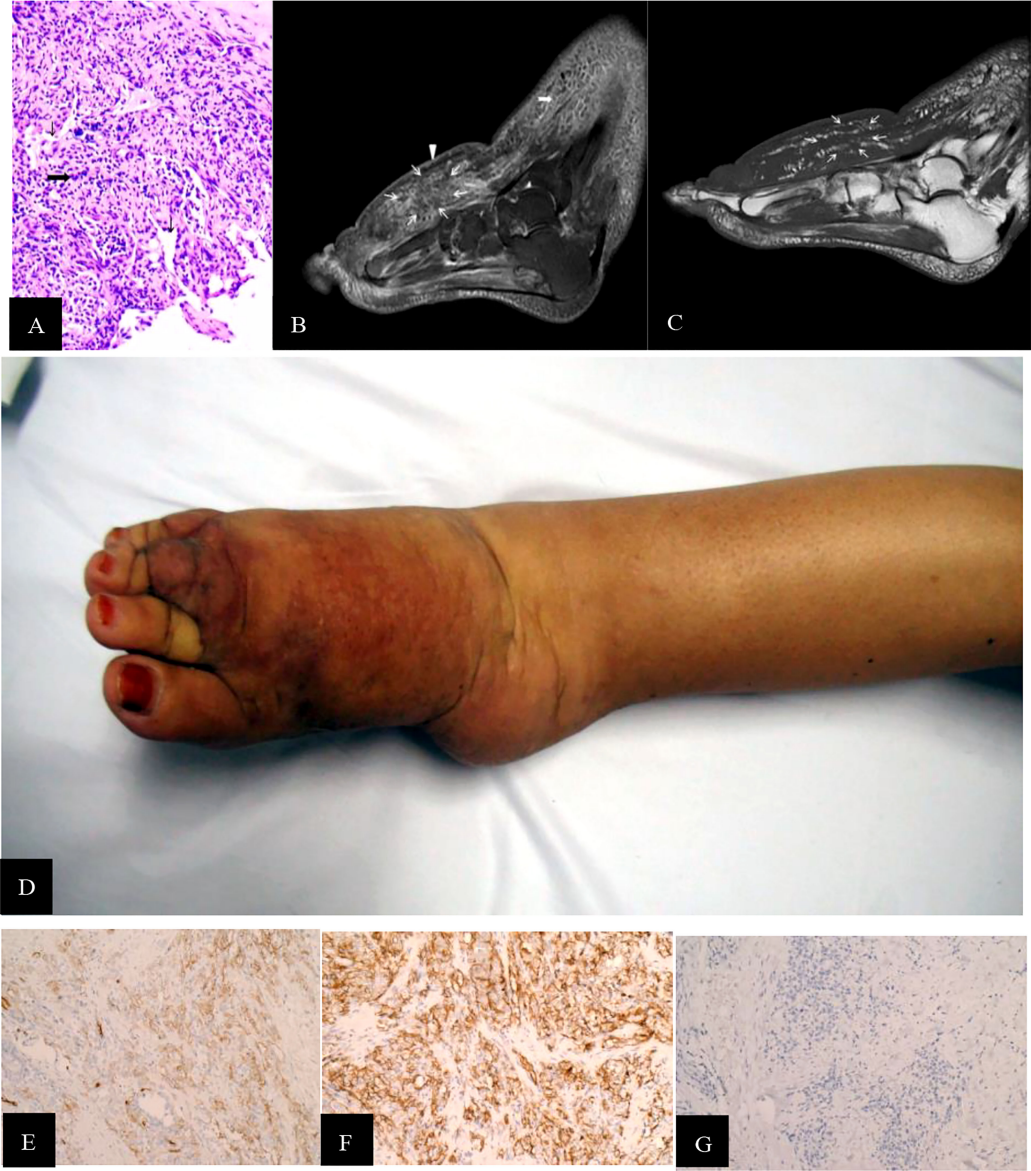
**(A)** STS PLE II type (histopathology, M200). Atypical epithelioid present as slit-like anastomosing spaces (fine black arrowhead) and are composed of loose spindle-type epithelioid cells (wide black arrowhead). There is a lack of vessels. The dysplastic collagen fibers show a scattered distribution. The underlying muscle had a normal signal intensity. **(B)** Sagittal T2WI was intermediately and heterogeneously decreased and scattered, revealing a patch-shape in the lymphedema (fine white arrowhead). If compared with the normal muscle tissues, the signal was slightly increased heterogeneously without any recognizable lymphedema signs. One of the regular signs of lymphedema, a Honeycomb sign, presented in the front subcutaneous tissue (wide white arrowhead). **(C)** Corresponding T1WI signal of STS was slightly decreased compared with the normal muscle (fine white arrowhead). **(D)** The dorsal skin and subcutaneous soft tissue of the right foot, which showed dark-red spots, increased skin temperature, and non-ulcer. **(E, F)** CD31 and CD34staining were positive (magnificatin, x200). **(G)** D2-40 staining was partially positive (magnification, x200).

Two tumor signs were recognized by MRI: a mass shape (classified into STS-PLE I type) and “trash ice” sign (classified into STS-PLE II type). The average survival time of the STS-PLE I type (**Figure 1D**) and the STS-PLE II type (**Figure 2D**) was 17.3 months and 54.5 months, respectively. The outline of the STS-PLE I type and the STS-PLE II type was clear and indistinct, respectively.

There was a recognizable difference in T2WI signals between the STS-PLE I type and the STS-PLE II type. The STS-PLE I type lesions showed a heterogeneous increase in signal intensity in T2WI (**Figure 1B**), while those of the STS-PLE II type exhibited a slightly or moderately reduced signal in T2WI (**Figure 2B**). There was no significant difference in the intensity of the signal on T1WI, which presented as a slightly or moderately decreased signal (**Figures 1C**, **2C**). There was no significant difference in the range of invasion, as the tumors invaded more than two locations.

### 3.3 Pathological results

For the STS-PLE I type, the histopathological characteristics were as follows. The tumor was predominantly composed of atypical epithelioid cells displaying hyperchromatism. Spindle endothelial cells lined the immature vessels. The tumor-adjacent tissue was rich in collagen fiber (**Figure 1A**). The histopathological characteristics of the STS PLE II type were as follows. The atypical epithelioid cells displayed as slit-like anastomosing spaces. The tumor was composed of loosely spindle-shaped epithelioid cells. The tumor showed a lack of vessels. Dysplastic collagen fibers were distributed in a scattered pattern (**Figure 2A**).

The Ki-67 index of the STS-PLE I type (mean 70%) was nearly three times higher than that of the STS PLE II type (mean 26.75%). Patients presenting a Ki-67 index >40% died within 5 years, in which the shortest survival time of two patients was 2 months and 3 months, respectively. There was no significant difference in the expression of D2-40. The lower the Ki-67 index (< 16%) or the earlier the onset, the longer the survival, especially for the STS-PLE I type. The stronger positive expression of CD31 and CD34 was correlated with a shorter survival time. The expression of D2-40 was positive in almost all cases and the distribution of P53 was scattered and not associated with a prognosis (**Table 4**).

**Table 4:** Immunohistochemical results of the patients with STS-PLE.

Number	Ki-67 (%)	CD31	CD34 D2-40	
1	90	++	++	–
2	90	++	++	+
3	30	+	+	+
3	50	+	+	+
4	40	+	+	+
6	2	+	+	+
7	15	+	+	+

+, focal positive; + +, diffuse positive; - negative.

## 4 Discussion

STS is an extremely rare malignant tumor with poor prognosis. STS based on secondary chronic limb lymphedema is an irreversible consequence of postoperative care. The causes include infection, filariasis, and injury (described as secondary lymphedema), of which 90% occurs postmastectomy. Although usually associated with mastectomy, the term ‘Stewart–Treves syndrome’ can be broadly applied. However, STS also develops from primary chronic lymphedema. To date, almost all reports and clinical data published on STS have been associated with secondary lymphedema (STS-SLE), including the average LD. Time before the tumor occurs ranges from 3.25 to 13 years (5, 8), with MST reported as 19 months and a 5-year survival rate of 5–13.6% (5, 8). The incidence of STS varies between 0.07% and 0.45% in the arm (4, 5). However, there have been no previous relevant reports of STS in primary limb lymphedema (STS-PLE). Furthermore, MRI has not been reported in the lower limb.

### 4.1 Clinical data in STS-PLE

In our study, the average survival rate of LD and MST at 5 years was 25.4 years and 45 months in 28.6% (2/7) of patients, respectively. The average LD and MST of STS-PLE were longer than those of STS-SLE (5, 8), by at least two-fold. Interestingly, MST in male patients (17.3 months) was three times shorter than that in female patients (54.5 months). The prognosis for male patients has not previously been described to be worse than that for female patients. The 5-year survival rate of STS-PLE was consistent with previous STS-SLE reports (5, 8). We speculate that it is possible that female patients with a better prognosis than male patients could be positively related with female sex hormone levels, that is, female sex hormone may act as a protective factor.

In our study, the STS-PLE location was found to be significant in the distal part of the limb (6/7). This finding was significantly different from previous STS-SLE reports (3–8). We consider it possible that the distribution trend is related to the two following factors: 1) the reduced lymphatic circulation was poorly correlated with gravity and 2) the frequency of immunodeficiency is higher (11). As a result, mutations in tumor cells should more likely occur in the distal part of the lower extremity.

In terms of skin findings, positive results (skin protuberance, dark red spots, and abnormally increased temperature) were observed in six cases. A young female patient presented with negative skin findings. If skin findings are present in PLE, especially with ulcer or hemorrhagic discharge, the diagnosis of STS should be considered.

The ratio between wide excision and amputation was 4:3, in which the MST of the former (34.75 months) was nearly 9 months less than the latter (43.67 months). In our study, there was a trend showing that the greater the scope of surgical resection or the earlier the operation, the better the prognosis (3–6). This finding might be helpful for clinical decision-making.

### 4.2 STS-PLE magnetic resonance findings compared with histological results

MRI is a useful tool to establish a modality for exploring the accurate anatomical definition and classification of peripheral lymphedema (6–8). There are few previous imaging reports of STS-SLE evaluated by computed tomography (12) and MRI (8, 13–15). An MRI study of STS-PLE has not been previously reported.

In our study, there were two different types of MRI findings. One was a mass shape (STS-PLE I type) observed in three male patients, and the other was the ‘trash ice’ sign d (STS-PLE II type) detected in four female patients. There was a discernible shape difference based on sex. The average survival time of STS-PLE I type (17.3 months) was three times shorter than that of STS-PLE II type (54.5 months). For the STS-PLE I type, the older the patient or the later the lymphedema occurred, the shorter the survival. However, there was no significant difference in STS-PLE II type. It seemed that the shape of the tumor on MRI was related to the prognosis. The survival of the STS-PLE I type was worse than that of the STS-PLE II type. Although there was no significant difference in the scope of invasion between the two types, MRI revealed tumor cell infiltration beyond the subcutaneous tissues, including the abnormally thickened dermis and superficial fascia adjacent to the tumor, as well as the disappearance of regular lymphedema signs referring to tumor nests distributed in the subcutaneous tissues (6–8).This finding may be helpful in surgical decision-making.

Stewart and Treves (1) first used the term lymphangiosarcoma (LAS) as an independent pathological diagnostic term. In 2011, when Yu and Yang (16) clearly explained the pathological diagnostic requirements of pure LAS and the mechanisms of angiosarcoma (AS), LAS was no longer used as a diagnostic term. In our study, we found that tumor shape and distribution could be easily recognized by the change in the T2WI signal (**Figures 1B**, **2B**), especially for the STS-PLE II type. The STS-PLE I type signal was heterogeneously decreased based on a background of lymphedema. With reference to signals of normal muscle tissue, the STS-PLE I type signal was intermediately increased heterogeneously on T2WI. The STS-PLE II type signal showed a slight and heterogeneous decrease and a scattered patch shape in lymphedema. With regard to the signal of normal muscle tissue, the STS-PLE II type signal showed a slightly increased heterogeneous in T2WI (6–8).

The histopathological findings revealed STS-PLE tumor cells were predominantly composed of dense atypical epithelioid cells with oval nuclear contours, prominent nucleoli, and eosinophilic cytoplasm in the two types (**Figures 1A**, **2A**). In the STS-PLE II type, collagen fibrils were more lavishly distributed between tumor nests than in the STS-PLE I type (**Figure 2A**). Otherwise, in the STS-PLE I type, there were many dilated clefts and channels with free erythrocytes. Moreover, several immature vessels in the tumor nests and collagen fibrils were distributed in the STS-PLE II type (**Figure 1A**). According to the above findings, we considered two factors could explain the correlation of both histopathological results and T2WI signal levels: 1) the higher the tumor cell-density and the more fibrous the stroma were, the lower the T2WI signal intensity, and 2) the richer proportion of immature and small vessels in STS-PLE, the higher T2WI signal intensity. Similar results (8, 13, 14) were found in the previous STS-SLE reports.

### 4.3 Immunohistochemical results and survival time in STS-PLE

CD31 and CD34 were used as specific vascular endothelial markers and D2-40 was used as an available selective marker for the lymphatic endothelium in immunohistochemistry assay. Yu and Yang (16) considered that the typical pure LAS should meet the following criteria: CD31 and CD34 being weakly positive or negative and D2-40 being positive. In our study, CD31 and CD 34 were positive in all cases. This finding meant that no cases were classified as pure LAS. One of seven patients exhibited negative expression of D2-40, and CD31 and CD34 showed strongly positive. We classified it as pure AS. Further, six out of seven cases were classified to a subset of LAS according to the previous reports (6–8, 16–19).

In our study, the expression of CD31 and CD34 was strongly positive in two male patients. In another five cases, there was no significantly different expression of CD31, CD34, and D2-40 (**Figures 1E–G**, **2E–G**). Of all the seven cases, those having a Ki-67 index of more than 40% died within 5 years, in which the shortest survivals reported were 2 months and 5 months. We found that irrespective of STS-PLE I type or STS-PLE II type, all cases shared the following basic features: 1) the lower the Ki-67 index (<16%) and the younger the patient, the longer the survival was, especially in the case of STS-PLE I type; 2) the stronger the positive expression of CD31, CD34, c-myc, and ERG were, the shorter the ST was; and 3) The expression of D2-40 was positive in nearly all cases and the distribution of P53 was scattered, so it could not show association between both types. A similar finding was reported previously (8).

Of course, when considering the prognostic factors of STS-PLE in this study, we only evaluated clinical data: MRI findings, pathological results, and immunohistochemical results. Notably, two other factors are easily ignored in clinical practice (17–20). One is the correlation between the evolution of lymphedema and the reduced surveillance of the immune system. The other is the correlation between the occurrence of local immune deficiency and evolution of STS-PLE. Although we did not include these two factors in our study, we should pay continuous attention to such issues in future research.

## 5 Conclusion

Pathological histopathological findings are closely related to MRI signs. Against the background of dense tumor cells, the richer the lumen of immature vessels and clefts, the higher the T2WI signal on the MRI (taking muscle signal as the internal reference standard), the worse the prognosis, and vice versa. In the adolescent age group, a tumor nest finding often shows the “trash ice” sign (STS-PLE II type) in a PLE, while in middle-aged and older patients, tumor nests show a mass shape (STS-PLE I type). The indicators (CD31, CD34, D2-40, and KI-67) of pathological immunohistochemistry are significantly correlated with clinical prognosis, with Ki-67 being especially prominent.

## Data availability statement

The original contributions presented in the study are included in the article/supplementary material. Further inquiries can be directed to the corresponding authors.

## Ethics statement

The study was approved by Human Research Ethics Committee at Beijing Shijitan Hospital of Capital Medical University and the procedures followed were in accordance with the ethical standards of the responsible committee on human experimentation (institutional or regional) and with the Helsinki Declaration of 1975, as revised in 2000. Written informed consent from the [patients/participants OR patients/participants legal guardian/next of kin] was not required to participate in this study in accordance with the national legislation and the institutional requirements.

## Author contributions

BL, JL, and KH performed the experiment and collected, analyzed, and drafted the manuscript. JL and YJ interpreted the data involved in the study and preprocessed image data. YJ and JM designed the study and ensured the questions related to all aspects of the work. YJ, JM, and XD contributed equally to this work and share corresponding authorship. All authors contributed to the article and approved the submitted version.
